# A Unique Case of Mycophenolate Induced Colitis after 10 Years of Use

**DOI:** 10.1155/2016/3058407

**Published:** 2016-09-07

**Authors:** Abhinav Goyal, Moiz Salahuddin, Yogesh Govil

**Affiliations:** Einstein Medical Center, Philadelphia, PA, USA

## Abstract

A 31-year-old female with a history of lupus nephritis on Hydroxychloroquine, Prednisone, and Mycophenolate Mofetil (MMF) for 10 years presented to the hospital for ankle swelling. On day four, she started to have severe, nonbloody, watery diarrhea with abdominal distension and tenderness. Stool PCR was negative for* C. difficile.* CT abdomen/pelvis showed gaseous distension of the colon without any obstruction. Flexible sigmoidoscopy revealed a normal looking mucosa. Histopathology showed crypt atrophy and increased crypt apoptosis, consistent with MMF colitis. The diarrhea resolved three days after stopping MMF. Although generally well tolerated, diarrhea is a common side effect of MMF. Most cases occur in the first six months of starting MMF. This case is unique because it describes MMF colitis in lupus after more than 10 years. Thus, MMF colitis should be considered as a differential in patients taking it, regardless of the duration of use.

## 1. Introduction

Mycophenolate Mofetil (MMF) is one of the most widely used immunosuppressants in solid organ transplant recipients. It is also being used increasingly as a part of steroid sparing regimen for various autoimmune diseases like systemic lupus erythematosus (SLE), especially in patients with lupus nephritis [1–3]. Mycophenolate is an inhibitor of Inosine Monophosphate Dehydrogenase which prevents T- and B-cell proliferation by preventing* de novo* Guanosine synthesis. Although generally well tolerated, diarrhea is one of the common adverse effects of the drug. It often leads to the patient having a colonoscopy given the immunocompromised status of these patients [4, 5]. While MMF induced colitis and diarrhea are a well-defined identity, the timing of diarrhea/colitis from the onset of MMF therapy remains uncertain [4]. Although most cases occur in the first six months after starting therapy in transplant patients, the longest duration between onset of diarrhea and start of MMF therapy was seen in a heart transplant recipient who presented 13 years after transplant [4, 6]. There are limited reported cases of MMF colitis presenting after more than 10 years, and none of them are SLE patients. We present a case of MMF colitis with SLE after more than 10 years of starting MMF therapy.

## 2. Case

A 31-year-old Caucasian female with a history of SLE and lupus nephritis on Hydroxychloroquine, Prednisone, and Mycophenolate Mofetil (uninterrupted) for ten years and chronic kidney disease stage IV presented to the hospital with complaints of bilateral ankle swelling for 2 days. She was found to have acute-on-chronic kidney injury; however her c-reactive protein and complement levels were normal and no other joints were involved. Her kidney function gradually improved and returned to baseline. On day four of her hospital stay, she started to have severe nonbloody, nonmucoid, watery diarrhea with up to 10 bowel movements per day. Over the next day she developed diffuse abdominal pain with abdominal distension but did not have any nausea or vomiting. On examination, she had generalized abdominal tenderness without guarding and normal bowel sounds. No significant change was noticed in her labs including white cell count, differential count, or liver function tests. The initial study of stool samples was negative for* Clostridium difficile* toxin by PCR based assay. PCR for Cytomegalovirus DNA was also checked to assess alternative causes of diarrhea and was negative.

Due to the concerning nature of her pain, a CT scan of her abdomen and pelvis was done which showed gaseous distension of ascending, transverse, and descending colon without wall thickening. An abrupt under distension with circumferential wall thickening of sigmoid colon without a clear cutoff was seen without any evidence of intestinal obstruction. Due to the high clinical suspicion for* Clostridium difficile* infection, she was started on oral Vancomycin and was planned for a flexible sigmoidoscopy the following day. The flexible sigmoidoscopy revealed a normal looking mucosa without any evidence of pseudomembrane or signs of inflammation. However, several random biopsies were taken. The histology showed focal active colitis with crypt atrophy and increased crypt apoptosis. These findings were found to be consistent with Mycophenolate Mofetil induced colitis. The Mycophenolate Mofetil was stopped and the patient's diarrhea resolved completely on the third day after stopping MMF. She was discharged home on Hydroxychloroquine and Prednisone for her lupus.

## 3. Discussion

MMF is usually a well-tolerated drug compared to other immunosuppressive medications but is commonly associated with gastrointestinal side effects, with the most common being diarrhea. The exact mechanism of this action is not known but some of the postulates are the blocking of* de novo* purine synthesis in enterocytes, acyl glucuronide metabolite induced toxicity, and antibacterial effect of MMF [4, 7–9].

In a patient on immunosuppressive medications especially in a patient with SLE presenting with diarrhea, there are several differential diagnoses that must be considered before MMF colitis can be diagnosed. Given the immunocompromised status of the patient, an infectious etiology is an important consideration, including unusual infections like CMV colitis. A colonoscopy with biopsy is often required for diagnosis.

**Figure 1:**
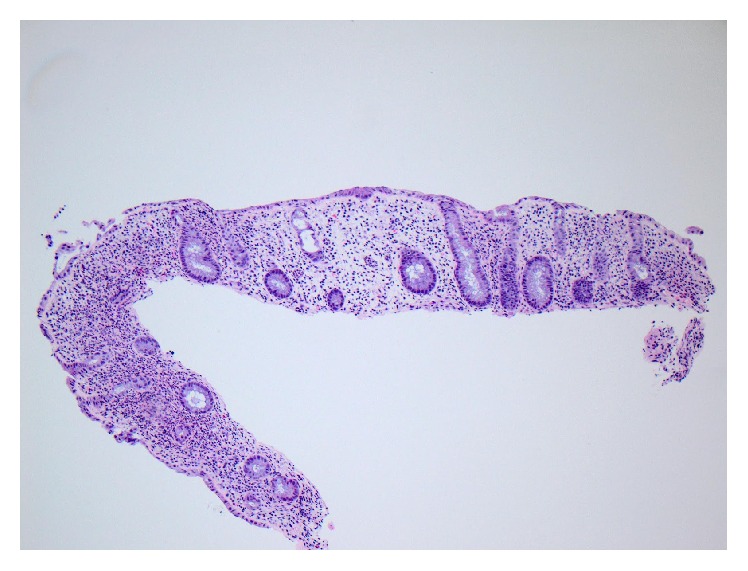
The colonic biopsies show crypt distortion with angulation, lamina propria edema, and increased lamina propria inflammation (H&E stain, 10x).

The most common colonoscopic appearance in MMF colitis is a normal looking mucosa [4]. However, there is a spectrum of histological changes on colon biopsy that has been associated with MMF colitis. These range from more common nonspecific colitis-like changes (31–50%) to inflammatory bowel disease- (IBD-) like changes (25–36%), graft versus host disease- (GVHD-) like pattern (8–19%), normal or near normal pattern (18–31%), and ischemia-like changes (3–12%) [4, 10, 11]. Liapis et al. found in their study that crypt distortion and increased apoptosis (as seen in our patient) constituted the main features; their degree and combination lead to either an appearance similar to IBD or a GVHD-like pattern [10–12]. Given that our patient did not have a history of allogeneic stem-cell transplantation, the finding of crypt apoptosis and atrophy (Figure 1) is characteristic of MMF induced colitis [12].

The temporal relationship between the onset of diarrhea and the duration of MMF therapy has not been well studied. The only literature available on the subject comes from a few retrospective studies, mostly on patients with solid organ transplant. De Grande et al. studied 16 kidney transplant patients and found that the mean onset of diarrhea was 44.3 ± 33.5 months. Bandelier et al. did a study assessing 11 patients with lupus being treated with MMF. One of their 11 patients developed a colitis resulting in discontinuation of the medication [13]. Most of the data that exists studying MMF is for transplant patients, and one of the risk factors was found to be the type of organ transplanted, with highest incidence in renal transplants [4, 12]. Most studies reported short-term outcomes and evaluated patients for only a few years after transplant. MMF is being increasingly used for autoimmune diseases such as lupus due to improved side effect profile compared to older drugs such as cyclosporine. Limited data currently exists on colitis in lupus patients on MMF therapy, and no significant long-term follow-up studies have yet been done to assess this.

There is no conclusive data regarding monitoring of MMF levels for therapeutic or adverse effects. Our patient had been on MMF for the last ten years continuously and had tolerated it well up to the present event. It is possible that worsening of her kidney function may have led to accumulation of MMF and thus resulted in toxicity. As it is not a common or recommended practice to measure MMF levels, most institutions including ours do not provide measurements of MMF levels. MacPhee et al. found that renal failure prolongs the half-life of the glucuronide and accumulation of the mycophenolic acid glucuronide metabolite [14]. However, there has been no study correlating MMF levels and side effects.

In conclusion, we present a case of diarrhea due to MMF induced colitis in a patient with SLE. This case reminds us that Mycophenolate induced diarrhea should be a part of a clinician's arsenal of differentials, regardless of the duration that the patient has been taking MMF for. It needs to be thought of as an idiosyncratic reaction and discontinuation of the drug seems to be the only effective therapeutic option available at this time.
